# The Add-On Effect of Solifenacin for Patients with Remaining Overactive Bladder after Treatment with Tamsulosin for Lower Urinary Tract Symptoms Suggestive of Benign Prostatic Obstruction

**DOI:** 10.1155/2010/205251

**Published:** 2010-10-26

**Authors:** Naoya Masumori, Taiji Tsukamoto, Masahiro Yanase, Hiroki Horita, Masaharu Aoki

**Affiliations:** ^1^Department of Urology, Sapporo Medical University School of Medicine, 060-8543 Sapporo, Japan; ^2^Department of Urology, Sunagawa City Hospital, 073-0196 Sunagawa, Japan; ^3^Department of Urology, Saiseikai Otaru Hospital, 047-0044 Otaru, Japan; ^4^Department of Urology, Kushiro Red Cross Hospital, 085-8512 Kushiro, Japan

## Abstract

*Objectives*. To investigate the add-on effect of solifenacin for Japanese men with remaining overactive bladder (OAB) symptoms after tamsulosin monotherapy for lower urinary tract symptoms (LUTS) suggestive of benign prostatic obstruction (BPO) in real-life clinical practice. *Methods*. Patients aged ≥ 50 having remaining OAB symptoms (≥ 3 of OAB symptom score (OABSS) with ≥2 of urgency score) after at least 4 weeks treatment by 0.2 mg of tamsulosin for BPO/LUTS received 2.5 or 5.0 mg of solifenacin for 12 weeks. The International Prostate Symptom Score (IPSS), QOL index and OABSS, maximum flow rate (Qmax) and postvoid residual urine volume (PVR) were determined. *Results*. A total of 48 patients (mean age 72.5 years) completed the study. There were significant improvement in IPSS (15.1 to 11.2) and QOL index (4.2 to 3.0) by add-on of solifenacin. Although the IPSS storage symptom score was significantly improved, there were no changes observed in the IPSS voiding symptom score. The OABSS showed significant improvement (8.0 to 4.8). No changes were observed in Qmax and PVR. *Conclusions*. Under the supervision of an experienced urologist, the additional administration of solifenacin to patients with BPO/LUTS treated with tamsulosin, is effective in controlling remaining OAB symptoms.

## 1. Introduction

Overactive bladder (OAB) symptoms are commonly observed in men with lower urinary tract symptoms (LUTS) suggestive of benign prostatic obstruction (BPO) [1]. Since *α*1-blocker monotherapy is efficacious in improving voiding symptoms and to a certain extent OAB symptoms [2, 3], it is recommended as the first line treatment for men with BPO + OAB [4]. However, remaining OAB symptoms are sometimes experienced, and such symptoms continuously impair quality of life (QOL).

To control OAB symptoms in men with BPO/LUTS, there are four strategies for medical treatment: administration of anticholinergic agent as the first line treatment [5], replace *α*1-blocker with anticholinergic agent [6], the combination of *α*1-blocker and anticholinergic agent as the first line treatment [7–10], and additional administration of anticholinergic agent [1, 11, 12]. According to the treatment recommendations proposed by the 6th International Consultation on New Developments in Prostate Cancer and Prostate Diseases [13], *α*1-blocker and anticholinergic combination therapy is recommended as a first line treatment for men with coexisting bladder outlet obstruction (BOO) + OAB symptoms. However, when we consider the promising efficacy of *α*1-blockers on OAB symptoms in men with BPO and adverse events as well as increased medical cost of anticholinergic agents, the add-on of anticholinergic agent following *α*1-blocker monotherapy seems to be clinically practical and acceptable.

Although several well-designed trials have indicated the add-on efficacy and safety of anticholinergic agents such as tolterodine [1, 11] and solifenacin [12], it remains unknown whether the evidence derived from the trials, most of which were performed in western countries, with strict inclusion and exclusion criteria can be applicable to elderly Japanese patients who have past illness and comorbidity with medication with real-world medication.

Tamsulosin hydrochloride having higher selectivity for *α*1A receptor subtype is one of the most frequently used *α*1-blockers in Japan since 1993. The approved dosage of tamsulosin is 0.2 mg/day in Japan. Solifenacin succinate launched in 2006 is the first available anticholinergic agent having higher selectivity for M_3_ receptor subtype approved for patients with OAB symptoms in Japan [14]. However, its efficacy and safety for men with BOO have not been demonstrated yet. In this study, we prospectively investigated the add-on effect of solifenacin for Japanese men with remaining OAB symptoms after monotherapy with *α*1-blocker, tamsulosin in real-life clinical practice.

## 2. Patients and Methods

Since this multi-institutional study was conducted in a real-life clinical practice setting, no strict inclusion criteria were applied. Between January 2008 and June 2009, patients aged 50 or older who reported that they were bothered by remaining OAB symptoms even after at least 4 weeks of treatment by 0.2 mg of tamsulosin hydrochloride once a day for LUTS suggestive of BPO were candidates for the study. Remaining OAB symptoms were defined as 3 or higher of the sum score of overactive bladder symptom score (OABSS) with 2 or higher of question 3 (urgency) score [15].

Indication of additional administration of solifenacin succinate was clinically decided based on experience of each urologist by concerning comorbidity, maximum flow rate (Qmax), postvoid residual urine volume (PVR), and so forth to minimize development of acute urinary retention. Solifenacin, 2.5 or 5.0 mg once a day (the dosage was selected by the physician based on the patient's age and comorbidity), was given for 12 weeks. During the study period, a change in type and dosage of *α*1-blocker was not allowed. The International Prostate Symptom Score (IPSS), QOL index, OABSS, Qmax, and PVR were determined before and after treatment. If there were adverse events observed, the severity, duration, and outcome were recorded.

Statistical comparisons of the mean values between before and after treatment of solifenacin were done using parametric paired *t*-test. For comparison between 2.5 mg and 5.0 mg of solifenacin, unpaired *t* test was used. *P* < .05 was considered statistically significant.

## 3. Results

### 3.1. Patient Population

Fifty-seven men were enrolled in the study. Analysis for safety was done using 52 patients because 5 men never come back to the hospital after the first administration of solifenacin. Of the 52, 48 patients completed the study and were provided for the efficacy analysis. Four patients were withdrawn because of adverse events. 

The age in the efficacy population was 72.5 ± 7.9 years old (mean ± SD). 54.2% of the patients had comorbidity such as hypertension (*n* = 19), diabetes mellitus (*n* = 5), history of cerebral infarction (*n* = 4), angina pectoris (*n* = 2), and others (*n* = 4) at the start of solifenacin. At baseline, average IPSS, QOL index, OABSS, Qmax, and PVR were 15.1, 4.2, 8.0, 10.8 ml/sec, and 16.6 ml, respectively. No patients had PVR greater than 100 ml. The average dosage of solifenacin given was 4.3 mg/day (31 patients: 5.0 mg/day, 17 patients: 2.5 mg/day). No patients changed the dosage of solifenacin during the study period. The patients having 2.5 mg were older (75.1 ± 5.7 years) than those having 5.0 mg of solifenacin (71.2 ± 8.6 years), although no statistical difference was observed (*P* = .068).

### 3.2. Efficacy Analysis

The IPSS and QOL index were significantly improved by the additional administration of solifenacin (Table 1). Although the storage symptom score (day frequency, urgency, and nocturia) and postmicturition symptom (incomplete emptying) were significantly improved, there were no changes observed in voiding symptom score (intermittency, weak stream, straining). OABSS and each symptom showed significant improvement by add-on of solifenacin (Table 2). No changes were observed in Qmax (10.8 ± 6.2 to 13.3 ± 9.9 ml/sec., *P* = .118, *n* = 36) and PVR (16.6 ± 23.3 to 15.8 ± 22.7 ml, *P* = .815, *n* = 45), although one patient showed significant increase of PVR from 62 to 246 ml. There were 32 patients with urge incontinence defined as 1 or greater of score of Q4 (urinary incontinence) in OABSS at baseline (32 of 48 men, 66.7%). Urge incontinence was disappeared in 18 patients (56.3%) by solifenacin treatment.

**Figure 1:**
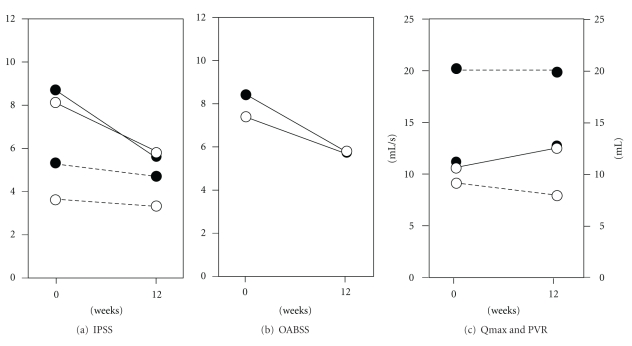
*Changes in parameters before and after 2.5 mg and 5.0* *mg of solifenacin treatment.* White and black circles indicated mean value of 2.5 mg and 5.0 mg of solifenacin, respectively. (a) The IPSS storage symptoms (line) and the IPSS voiding symptoms (dotted lines) 2.5 mg: *n* = 16, 5.0 mg: *n* = 32, (b) OABSS, 2.5 mg: *n* = 16, 5.0 mg: *n* = 32, and (c) Qmax (line, 2.5 mg: *n* = 12, 5.0 mg: *n* = 24) and PVR (dotted lines, 2.5 mg: *n* = 15, 5.0 mg: *n* = 30).

There were no differences in the patterns of changes in the IPSS storage symptoms (significant decrease), the IPSS voiding symptoms (no change), OABSS (significant decrease), Qmax (no change), and PVR (no change) between 2.5 mg and 5.0 mg of solifenacin (Figure 1). No statistical differences were observed in the amount of changes in the IPSS storage symptoms (−2.3 ± 1.9 versus −3.1 ± 2.8, *P* = .282), the IPSS voiding symptoms (−0.3 ± 2.6 versus −0.5 ± 3.1, *P* = .757), OABSS (−2.6 ± 2.3 versus −3.4 ± 3.0, *P* = .320), Qmax (2.1 ± 6.8 versus 2.2 ± 8.8, *P* = .977), and PVR (−1.3 ± 15.9 versus −0.5 ± 24.1, *P* = .891).

### 3.3. Safety Analysis

Of the 52 patients of the safety population, 9 adverse events were observed in 7 patients (13.5%). Of the 7, 2 (2 of 17: 11.8%) and 5 (5 of 35: 14.3%) received 2.5 mg and 5.0 mg of solifenacin, respectively. Constipation was the most frequent adverse event reported in 3 patients (5.8%), then dry mouth in 2 (3.9%), difficulty of voiding in 2 (3.9%), increased PVR in 1 (1.9%), and elevated liver enzyme in 1 (1.9%). Difficulty of voiding or increased PVR in 3 patients disappeared following termination of solifenacin during the study period. Solifenacin could be continued by the end of study period in the remaining 4 patients because adverse events were mild in degree. One patient (1.9%) quit taking solifenacin because of symptomatic worsening.

## 4. Discussion

*α*1-blocker is an efficacious treatment modality to reduce overactive bladder symptoms in patients with LUTS suggestive of BPO. According to the Japan-Tamsulosin I-PSS Survey [2] conducted as the nation-wide postmarketing surveillance of tamsulosin in 5,363 patients with BPO/LUTS, 12 weeks of tamsulosin treatment significantly improved the IPSS from 16.7 to 8.6 and the QOL index from 4.5 to 2.5. In the study, not only the IPSS voiding symptom score but also the IPSS storage symptom score were significantly improved from 7.1 to 3.4 and 7.2 to 4.1, respectively. Similar result was obtained when 50 mg of naftopidil was given to patients with BPO/LUTS [3]. However, persisting storage symptoms not responding to *α*1-blockers are frequently observed in a clinical setting [1]. In the subjects evaluated in our study, despite of administration of tamsulosin, the IPSS and QOL index were 15.1 and 4.2, respectively. The IPSS voiding symptoms seemed to be well controlled by tamsulosin monotherapy although no data before tamsulosin treatment were available. On the other hand, due to inclusion criteria to define remaining OAB symptoms, the IPSS storage symptom score remained high comparable to that before *α*1-blocker treatment [2, 3]. Thus, additional treatment is mandatory for BPO/LUTS patients with persisting OAB symptoms to further improve their QOL.

Remaining OAB symptoms are mainly caused by detrusor overactivity rather than BPO. There are several mechanisms to explain the highly frequent association of bladder overactivity with BPO/LUTS such as denervation hypersensitivity, modulated detrusor properties, increased release of urothelial neurotransmitters, and increased afferent stimulation from the urethra [4]. Since anticholinergic agents contribute to improve OAB symptoms through the blockade of muscarinic receptors on the smooth muscle, urothelium, and afferent nerves, they may be effective to control remaining OAB symptoms after *α*1-blockers monotherapy.

In the present study, additional administration of solifenacin for patients with BPO/LUTS treated with tamsulosin revealed significant improvement of the remaining OAB symptoms without deterioration of voiding symptoms, Qmax, and PVR. In addition, neither acute urinary retention nor severe adverse events were observed during the study period.

There are several well-designed prospective trials that investigate the add-on efficacy and safety of anticholinergic agent after *α*1-blocker monotherapy. Lee et al. [1] added 4 mg/day of tolterodine for 44 men with urodynamically proven BOO + OAB who failed 2–4 mg/day of doxazosin monotherapy for 3 months. Thirty-two men (73%) showed symptomatic improvement defined as a decrease in IPSS of >3 points by adding of tolterodine. Chapple et al. [11] conducted a randomized double-blind trial in men with remaining OAB symptoms (mean urinary frequency ≥8 times per 24 hours including ≥1 micturition-related urgency episode per 24 hours) with “some moderate” bladder-related problems on the Patient Perception of Bladder Condition (PBSC) after *α*1-blocker monotherapy (alfuzosin, doxazosin, or tamsulosin) at least for 4 weeks. A total of 652 men were randomly allocated into placebo + *α*1-blocker (*n* = 323) or tolterodine ER 4 mg + *α*1-blocker (*n* = 329) for 12 weeks. Although no significant difference in PBSC improvement as the primary end point was observed between the 2 groups, men with tolterodine ER + *α*1-blocker showed significantly greater improvements in storage symptoms such as urinary frequency, urgency, the IPSS storage symptom score, and OAB-q symptom bother scale. There were no clinically meaningful changes in Qmax and PVR. Kaplan et al. [12] reported the efficacy and tolerability of solifenacin add-on to men with residual urgency and frequency (mean urinary frequency ≥8 times per 24 hours including ≥1 urgency episode per 24 hours in a bladder diary) after 0.4 mg/day of tamsulosin for 4 or more weeks. A total of 398 men were randomized to 12 weeks of solifenacin 5 mg + tamsulosin (*n* = 202) or placebo + tamsulosin (*n* = 195). Although there were no significant differences in reductions of urinary frequency per 24 hours (−1.05 versus −0.67, *P* = .135) and the IPSS storage symptom score (−2.80 versus −2.33, *P* = .074) between the 2 groups, urgency episode per 24 hours was significantly reduced in the group with add-on of solifenacin (−2.16 versus −1.10, *P* < .001). Urinary retention was reported for 7 patients (3%) and required catheterization on solifenacin + tamsulosin whereas none was reported on placebo + tamsulosin. Thus, they concluded that solifenacin + tamsulosin was well tolerated although closer supervision may be required for men with severe BOO.

There are several studies that investigated the add-on effects of anticholinergic agents after *α*1-blockers monotherapy in Japan [16–22]. Although the studies investigated very small number of patients using various protocols in terms of type of proceeding *α*1-blockers (tamsulosin [17, 19], naftopidil, silodosin [21], combined [16, 20, 22]), required minimum duration of *α*1-blockers monotherapy by administration of anticholinergic agents (2 weeks [19], 4 weeks [16–18, 21, 22]), type of anticholinergic agents (propiverine [16], solifenacin [17–19], tolterodine [20], imidafenacin [21, 22]), and duration of add-on (2 weeks [21], 4 weeks [16, 18, 19], 8 weeks [17, 20, 22]), most of studies demonstrated similar results that the IPSS, QOL index, the IPSS storage symptoms, and OABSS were improved and the IPSS voiding symptoms remained unchanged by add-on of anticholinergic agents. No worsening of Qmax and PVR was observed except in one report [16]. In addition, no studies reported development of acute urinary retention. It is interesting that relatively consistent results were achieved even though each study had limited power to draw conclusion due to small numbers of patients with different type and dosage of *α*l-blockers and anticholinergic agents.

There is limited information on dosage of anticholinergic agent when the balance between the efficacy and safety is considered. It is reported that either 2.5 mg or 5.0 mg of solifenacin was effective and safe in the previous studies in [17–19]. In our study, there were no obvious differences in efficacy and safety between 2.5 mg and 5.0 mg of solifenacin, where dosage was decided based on experience of each urologist. Although there are no criteria to select appropriate candidates for the combination in the literature, age, general condition, comorbidity, degree of BPO estimated by prostate volume, Qmax, and PVR, and so forth may be considered when the indication and dosage of solifenacin were decided in our study. In fact, no patients with 100 ml or larger PVR were enrolled in the study despite of no definitive criterion for PVR.

Thus, add-on of anticholinergic agents is promising also in Japan, although a well-designed large-scale study is lacking. The prospective randomized study that investigated the add-on of 10 mg or 20 mg of propiverine for men who failed 0.2 mg of tamsulosin monotherapy for 8 weeks is now under preparation for publication [23].

There was limitation in this study. Since we did not evaluate the prostate volume just before add-on of solifenacin, some men might not have benign prostatic enlargement origin in LUTS. Thus, the efficacy and safety of solifenacin according to the prostate volume were unknown. 

In conclusion, under the supervision of an experienced urologist, the additional administration of solifenacin to patients with BPO/LUTS treated with *α*1-blockers is effective in controlling remaining OAB symptoms and in improving QOL. The efficacy and safety of add-on of solifenacin as well as selection criteria and appropriate dosage should be investigated in a large-scale randomized study.

## Figures and Tables

**Table 1:** Change in IPSS and QOL index before and after solifenacin treatment (*n* = 48).

Parameter	Before	12 weeks	*P*-value^(2)^
Incomplete emptying	2.0 ± 1.7^(1)^	1.3 ± 1.3	*P* < .01
Day frequency	2.7 ± 1.4	2.0 ± 1.2	*P* < .001
Intermittency	1.6 ± 1.7	1.4 ± 1.3	*P* = .516
Urgency	2.8 ± 1.5	1.5 ± 1.2	*P* < .001
Weak stream	2.3 ± 1.7	1.9 ± 1.5	*P* = .066
Straining	0.9 ± 1.2	0.9 ± 1.2	*P* = .644
Nocturia	3.0 ± 1.2	2.2 ± 1.2	*P* < .001
IPSS storage symptoms	8.5 ± 2.9	5.6 ± 2.6	*P* < .001
IPSS voiding symptoms	4.7 ± 3.7	4.2 ± 3.2	*P* = .253
IPSS total score	15.1 ± 5.9	11.2 ± 5.1	*P* < .001
QOL index	4.2 ± 1.2	3.0 ± 1.2	*P* < .001

^(1)^Mean ± SD. ^(2)^Paired *t*-test.

**Table 2:** Change in OABSS before and after solifenacin treatment (*n* = 48).

Parameter	Before	12 weeks	*P*-value^(2)^
Daytime frequency	0.9 ± 0.6^(1)^	0.6 ± 0.5	*P* < .05
Nighttime frequency	2.5 ± 0.7	2.0 ± 1.0	*P* < .001
Urgency	3.1 ± 1.0	1.5 ± 1.3	*P* < .001
Urgency incontinence	1.5 ± 1.4	0.7 ± 1.2	*P* < .001
OABSS	8.0 ± 2.5	4.8 ± 2.6	*P* < .001

^(1)^Mean ± SD. ^(2)^Paired *t*-test.
